# Mapping the Prosthetic–Host Interactome: From Systemic Inflammation to Biological Integration in Mesh-Enhanced Therapies METs—A Scoping Review

**DOI:** 10.3390/ijms27146153

**Published:** 2026-07-09

**Authors:** Florentina Cristina Finascu, Valentin Constantin Oprea, Mihai Toma, Carmen Elena Bucuri, Calin Molnar, Bogdan Andrei Finascu, Bianca Liana Grigorescu, Bogdan Andrei Suciu

**Affiliations:** 1 Doctoral School of Medicine, “George Emil Palade” University of Medicine, Pharmacy, Science and Technology, 540139 Targu-Mures, Romania; florentinacristina.scarlat@gmail.com; 2 Department of Surgery, “Iuliu Hatieganu” University of Medicine and Pharmacy, 400347 Cluj-Napoca, Romania; cbucurie@yahoo.com (C.E.B.); bogdan.finascu@yahoo.ro (B.A.F.); 3 Department of Surgery, “Constantin Papilian” Emergency Clinical Military Hospital, 400132 Cluj-Napoca, Romania; dr.mtoma.eru@gmail.com; 4 First Department of Surgery, Emergency Clinical County Hospital Targu Mures, 540136 Targu Mures, Romania; molnar.calin@yahoo.com (C.M.); suciubogdanandrei@yahoo.com (B.A.S.); 5 Department of Anaesthesia and Intensive Care, Emergency Clinical County Hospital Targu Mures, 540136 Targu Mures, Romania; bianca.grigorescu@umfst.ro

**Keywords:** inguinal hernia, Mesh-Enriched Therapies (MET), macrophage polarization, foreign body response, regenerative surgery, prosthetic–host interactome

## Abstract

Despite reducing hernia recurrence, synthetic meshes often trigger persistent foreign body responses (FBRs). Mesh-enriched therapies (METs), incorporating autologous cellular components (MSCs, PRP, SVF), can regeneratively reprogram the host-prosthetic interactome. Following PRISMA-ScR guidelines, this scoping review involved a systematic search of PubMed, Embase, and Scopus (2000–2025). We utilized the PCC (Population, Concept, Context) framework to map evidence across systemic inflammation, local FBR, and bio-augmentation strategies. A total of sixty-five studies were synthesized and categorized into three primary thematic pillars. Regarding the Systemic Response (*n* = 25), the data established a predictable “foreign body signature” characterized by prominent C-reactive protein (CRP) and interleukin-6 (IL-6) spikes within the first 48 h post-implantation. For the Local Foreign Body Reaction (FBR, *n* = 19), human explant data extending up to 180 months revealed a perpetual, immune-mediated state driven by matrix metalloproteinase-2 (MMP-2) matrix remodeling and the development of “bridging fibrosis.” Finally, concerning Mesh-Enriched Therapy (MET) Integration (*n* = 21), biological enrichment successfully shifted the M1/M2 macrophage ratio toward a pro-regenerative, CD163+/CD206+ phenotype. While MET consistently enhanced vascular endothelial growth factor (VEGF)-driven angiogenesis and optimized the Collagen I/III ratio, a notable 22.2% discrepancy rate across the literature underscores the critical need for precise transforming growth factor-beta 1 (TGF-β1) dosing and release kinetics to prevent hyper-fibrosis. MET shifts hernia repair from passive mechanical reinforcement to active “biocamouflage” and integration. By modulating the Th1/Th2 rheostat, enriched therapies mitigate chronic inflammation and long-term complications. Standardized clinical trials are essential to optimize the therapeutic window for hybrid integration.

## 1. Introduction

Groin hernia repair remains one of the most frequent yet controversial procedures in general surgery. While current standards favor tension-free, mesh-based techniques, including open (Lichtenstein) and minimally invasive—such as transabdominal preperitoneal (TAPP) and totally extraperitoneal (TEP)—approaches, the goal of achieving recurrence rates below 1% and chronic pain under 3% remains elusive [1,2,3]. The clinical success of these repairs is often hampered by “mesh-associated pathology,” which is characterized by chronic pain (10–30% of cases), infection, fibrosis, migration, and visceral erosion [4,5].

In the current state of the art, modern herniology relies heavily on synthetic polymers, primarily lightweight polypropylene (PP), expanded polytetrafluoroethylene (ePTFE), and various composite matrices designed to reduce mechanical profile and optimize structural porosity. Despite these physical advancements, these materials inherently lack biological signaling cues, failing to establish functional interfaces with the host side. Instead, these secondary complications are primarily driven by the Foreign Body Reaction (FBR), a complex immune sequence ranging from initial blood-material interactions to persistent chronic inflammation that culminates in the dense fibrotic encapsulation of the device [6,7].

Because hernia meshes are permanent and increasingly implanted in younger populations, the associated risks are lifelong. Notably, the 50th percentile for long-term adverse events occurs at 3.75 years post-implantation, with complications and risks persisting for over 15–17 years; consequently, short-term clinical studies may underestimate complications by up to 50% [8,9]. Despite the advanced mechanical evolution of prosthetics, biocompatibility remains the primary “thorny issue” of modern herniology. Mesh-enriched therapies (MET), involving the integration of autologous cellular components to “hide” the foreign material from the host’s innate immune system, represent a promising strategy to modulate this inflammatory response [10]. However, the available scientific evidence remains highly fragmented across various cell lines and seeding techniques. This scoping review aims to comprehensively map the systemic and local inflammatory landscape of hernia repair and evaluate the capacity of cellular therapies to foster true biological integration.

## 2. Material and Methods

### 2.1. Study Design and Protocol Registration

This scoping review follows the PRISMA-ScR (Preferred Reporting Items for Systematic Reviews and Meta-Analyses extension for Scoping Reviews) guidelines [11]. The methodology was grounded in the framework established by Arksey and O’Malley [12]. The study protocol was prospectively registered in the Open Science Framework (OSF) Registry on 15 March 2026 (Protocol ID: https://doi.org/10.17605/OSF.IO/T2QNX (accessed on 2 June 2026).

### 2.2. Search Strategy

A comprehensive search of the PubMed, Scopus, and Embase databases was performed to identify all relevant literature published between January 2000 and December 2025. The search string utilized a combination of Medical Subject Headings (MeSH) terms and free-text keywords, structured into three primary thematic blocks (Pathology, Device, and Therapy) combined via the Boolean operator AND.

Block 1 (Pathology): (“Hernia, Inguinal”[MeSH] OR “Hernia, Femoral”[MeSH] OR “Groin Hernia*”[Title/Abstract])

Block 2 (Device): (“Surgical Mesh”[MeSH] OR “Mesh*”[Title/Abstract] OR “Prosthesis”[Title/Abstract])

Block 3 (Therapy): (“Mesenchymal Stem Cells”[MeSH] OR “Cell- and Tissue-Based Therapy”[MeSH] OR “Stem Cell*”[Title/Abstract] OR “Stromal Vascular Fraction”[Title/Abstract] OR “Bio-synthetic”[Title/Abstract] OR “Cell-Enriched”[Title/Abstract] OR “MET”[Title/Abstract]).

To ensure reproducibility across different indexing systems, the comprehensive search strategies adapted for each individual database are provided in the Supplementary Materials (Supplementary Table S1).

No language restrictions were applied during the database interrogation. The references of identified articles were manually screened for additional relevant studies.

### 2.3. Eligibility Criteria

To guarantee clinical relevance, the PCC (Population, Concept, and Context) was applied:

#### 2.3.1. P (Population)

In vivo (animal models) or clinical human cohorts undergoing prosthetic mesh implantation for groin hernia repair.

#### 2.3.2. C (Concept)

Utilization of Meshed Enriched Therapies (MET), specifically evaluating the biological augmentation of synthetic or biologic scaffolds with viable cells—such as mesenchymal stem cells (MSCs), adipose-derived stem cells (ADSCS), stromal vascular fraction (SVF)—or autologous hemoderivatives.

#### 2.3.3. C (Context)

Any surgical repair approach (open or laparoscopic) evaluating local or systemic inflammation, structural tissue integration, secondary biomaterial complications, or clinical patient outcomes.

Only original, peer-reviewed full-text articles published in English were included. We excluded pediatric series, reviews, editorials, conference abstracts, and case reports. Additionally, studies focusing exclusively on hiatal hernias, or those lacking detailed methodological data were excluded. To ensure comprehensive coverage, the reference lists of all included articles and relevant gray literature (including 106 identified doctoral theses) were manually screened.

### 2.4. Data Selection and Synthesis

**Figure 1:**
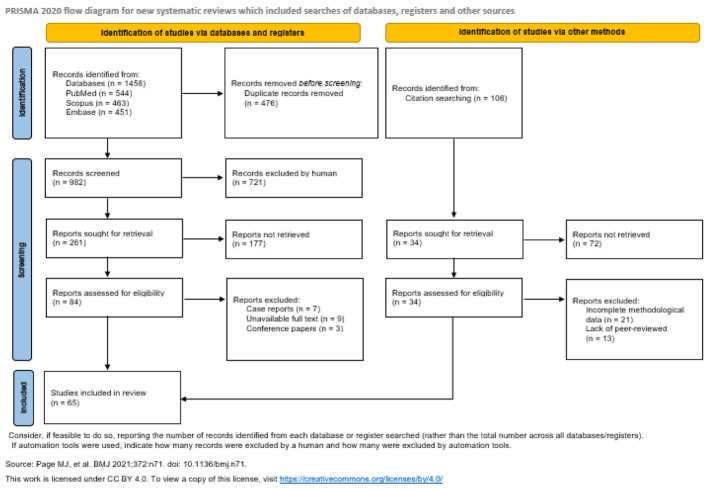
PRISMA flow chart of study selection (included and excluded) [13].

The screening process was conducted independently by two reviewers (F.F. and V.O), using a multistep approach. Initially, titles and abstracts were screened against the eligibility criteria, followed by a rigorous full-text assessment of all potentially relevant papers. Any discrepancies or disagreements between the primary screeners were resolved through consensus or consultation via a third senior reviewer (MT). The full study selection flow is visually summarized in the PRISMA flow diagram (Figure 1).

## 3. Results

### 3.1. Study Selection and Characteristics

The initial systematic database search identified 1458 records (PubMed: *n* = 544; Scopus: *n* = 463; Embase: *n* = 451). Following the removal of 476 duplicate records, 982 items underwent rigorous title and abstract screening, resulting in the exclusion of 721 irrelevant studies. Concurrently, a manual search of gray literature and academic repositories identified 106 theses. Of the 261 reports with thoughts for retrieval, 177 reports (including all 106 theses due to incomplete primary methodological data or lack of peer-reviewed experimental outcomes) were not retrieved.

Consequently, 84 articles underwent comprehensive eligibility assessment. After evaluating the full texts, 19 articles were excluded with specific reasons: case reports (*n* = 7), unavailable full texts (*n* = 9), and conference papers (*n* = 3). Ultimately, 65 original studies met all eligibility criteria and were included in the scoping review (Figure 1).

The included literature exhibited significant heterogeneity and was categorized into three primary thematic focus areas: the systemic inflammatory footprint (*n* = 25), local foreign body reaction and human explant dynamics (*n* = 19), and mesh-enriched therapies (MET) for biological integration (*n* = 21). The global methodological distribution, sample characteristics, targeted biomarkers, and primary biological endpoints of these 65 synthesized studies are systematically cross-referenced and unbundled in Table 1.

Methodological quality assessment using the JBI critical appraisal checklists for the clinical cohorts demonstrated a low risk of bias, with over 80% of the studies fulfilling the essential reporting parameters regarding patient selection and outcome measurements. For the preclinical in vivo literature, the SYRCLE risk of bias tool revealed moderate-to-high methodological quality; however, specific domains—particularly allocation concealment and reviewer blinding—were frequently underreported across 68% of the animal series (detailed in Supplementary Table S2).

### 3.2. The Systemic Immunological Footprint

The systemic inflammatory response following groin hernia repair was evaluated across 25 studies, encompassing a total cohort of 1222 patients. Structurally, this literature subset comprised 16 randomized controlled trials (RCTs), 3 prospective non-randomized trials, and 6 retrospective series [14,15,16,17,18,19,20,21,22,23,24,25,26,27,28,29,30,31,32,33,34,35,36,37,38]. Surgical interventions evaluated across these cohorts ranged from open tissue-based repairs to minimally invasive laparoscopic approaches. Synthetic polypropylene (PP) constructs were utilized in 23 of the 25 studies, establishing the primary biomaterial baseline for the baseline circulating immune response.

Across the analyzed literature, 43 unique systemic inflammatory and immunological biomarkers were investigated. Quantitatively, C-reactive protein (CRP) and Interleukin-6 (IL-6) were the most frequently tracked indicators, each reported in 18 separate studies (72%). These parameters were followed by White Blood Cell (WBC) counts in 10 studies (40%) and Tumor Necrosis Factor-alpha (TNF-α) in 6 studies (24%). The distribution of these study characteristics, biomaterial variations, and targeted biomarker frequencies across the entire synthesized literature is cross-referenced and integrated within the unified.

Collectively, the extracted data indicate that mesh implantation initiates a time-dependent systemic inflammatory cascade. The chosen surgical access route and the specific surface properties of the implanted biomaterials primarily modulate the overall magnitude and temporal kinetics of this circulating immune response.

Surgical techniques ranged from tissue-based repairs to laparoscopic approaches. Notably, 23 of the 25 studies utilized Polypropylene (PP), establishing a consistent baseline for the “foreign body signature” in the circulatory system.

### 3.3. Local Foreign Body Dynamics and Human Explant Analysis

The local foreign body reaction (FBR) and tissue remodeling interfaces were evaluated across 19 studies [39,40,41,42,43,44,45,46,47,48,49,50,51,52,53,54,55,56,57,58] utilizing three distinct experimental models. Human explant series (*n* = 4 studies) provided retrospective data on 743 recovered meshes explanted between 2 and 180 months due to recurrence, chronic groin pain, or mesh infection. Experimental in vivo animal models (*n* = 13 studies)tracked responses in 674 animal subjects (rats, rabbits, and mice) over a timeline extending from 1 day to 26 weeks. Early-stage cell response kinetics were evaluated via in vivo and ex vivo assays (*n* = 2 studies) utilizing human peripheral blood and rat kidney fibroblasts.

Tissue integration was quantified across these models using histological scoring and PV parameters. The preclinical in vivo series demonstrated rapid formation of organized foreign body granulomas. Infiltration kinetics systematically shifted from an acute neutrophilic phase between days 1 and 7 dominated by host fibroblasts and T-lymphocytes. This cellular persistence was corroborated by the long-term human explant data, indicating that the local immune response remains chronically active.

Quantitative analysis of host-mesh microenvironment highlighted three primary molecular axes modulating the long-term fate of the device:

1. Matrix remodeling: sustained local inflammatory signaling, driven by Tumor Necrosis Factor-α (TNF-α) directly correlated with elevated Matrix Metalloproteinase-2 (MMP-2) expression (*p* < 0.05). In chronic human explants, over 50% of the infiltrating macrophage population co-expressed MMP-2, confirming an active, prolonged state of extracellular matrix (ECM) turnover. Morphometrically, the partial volume of inflammation (PV-I) peaked sharply between days 3 and 7 in animal models before gradually declining, while remaining higher in heavyweight (HW) constructs compared to lightweight (LW) designs. Concurrently, the partial volume of connective tissue (PV-CT) increased significantly from day 7 to day 28 (*p* < 0.01, with small-pore constructs showing aggressive bridging fibrosis compared to large-pore, class 1 meshes.
2. Macrophage polarization: a critical biological transition from pro-inflammatory M1 phenotypes to pro-fibrotic M2 phenotypes occurred predominantly between days 7 and 21 in animal studies. This phenotypic shift dictates collagen deposition subsequently. Long-term human explant analyses demonstrated that while M1 macrophages dominate the acute phase, M2 macrophages sustain chronic, late-stage fibrotic responses. Furthermore, a highly significant positive correlation was identified between CD68+ macrophages and CD3+ T-lymphocytes with very late-term human explants (r = 0.341, *p* = 0.001), proving the existence of a persistent adaptive immune response at the material interface.
3. Metabolic flux: cellular turnover at the interface—quantified via Ki67 proliferation indices and TUNEL apoptosis assays—demonstrated a sharp metabolic peak between days 7 and 21 post-implantation. While this cellular birth-and-death flux stabilized after 90 days in small animal models, metabolic activity remained significantly elevated above native baseline thresholds, directly reflecting the ongoing host response to the mechanical and chemical surface properties of the polymer filaments.

### 3.4. Biological Augmentation Strategies: Mechanistic Synthesis

The scoping review identified 21 eligible studies [59,60,61,62,63,64,65,66,67,68,69,70,71,72,73,74,75,76,77,78,79] investigating the biological augmentation of synthetic surgical scaffolds. This literature subset primarily comprised preclinical in vivo models (*n* = 19, evaluating 514 animal subjects including rats, pigs, rabbits, goats, rams, and dogs) and initial clinical translation series (*n* = 2, encompassing 116 patients, of whom 34 received advanced biological enrichment). The therapeutic interventions were stratified into three primary categories based on the enrichment substrate: blood-derived components (*n* = 12, utilizing platelet-rich plasma [PRP] or platelet-rich fibrin [PRF]), cell-based therapies (*n* = 7, utilizing mesenchymal stem cells [MSCs]), adipose-derived stem cells [ADSCs], or stromal vascular fraction [SVF], and protein-based scaffolds or hybrid configurations (*n* = 2, utilizing fibrin or combination protocols). All baseline characteristics, model distributions, and targeted biomarkers from these 21 studies are fully integrated into the master repository in Table 1.

Local angiogenesis was consistently enhanced across all evaluated enrichment categories. The application of autologous blood-derived secretomes (PRP and PRF) triggered a significant upregulation of Vascular Endothelial Growth Factor (VEGF), which directly correlated with increased microvessel density quantified via CD31 and CD34 immunohistochemical staining. Across the preclinical animal series, mRNA expression normalization using Glyceraldehyde 3-phosphate dehydrogenase (GAPDH) ensured analytical consistency in evaluating these growth factor surges and tracking collagen isoform expression.

This biological augmentation significantly accelerated the local extracellular matrix (ECM) remodeling cascade, driving a rapid transition from disorganized acute granulation tissue to a stabilized fascial matrix. This structural maturation was characterized by an increased Collagen Type I to Type III ratio (Collagen I/III), which serves as a reliable histological proxy for enhanced structural tensile strength. Concurrently, a marked upregulation of alpha-smooth muscle actin (*α-SMA*) reflected the targeted recruitment and activation of functional myofibroblasts, which orchestrated the development of a mature, organized cicatrix while mitigating clinical mesh shrinkage. Quantitative biomechanical evaluations (including Young’s Modulus and load-to-failure parameters) confirmed that mesh-enriched therapies (*MET*) significantly improved the structural elasticity and mechanical load capacity of the abdominal wall repair, facilitating a more physiological strain distribution.

High-resolution local cytokine profiling revealed that biological enrichment establishes a balanced *IL-6/IL-10* cytokine axis, successfully modulating the initial post-surgical immune response. A pivotal outcome of stem cell (MSC and SVF) enrichment was the direct polarization of host macrophages away from a chronic, aggressive M1 phenotype and toward a pro-regenerative, reparative M2 phenotype. This phenotypic shift, tracked via specific M1/M2 ratios and validated using stem cell immunophenotyping markers (positive expression of CD90 and CD44, coupled with negative CD45 lineage expression), effectively mitigated dense fibrotic encapsulation and foreign body giant cell fusion. Furthermore, adaptive immune profiling indicated a localized Th2-dominant shift, promoting immune tolerance and reducing host rejection of the synthetic mesh filaments.

A clear translational correlation was identified between the expression pathways mapped in preclinical research and the clinical outcomes documented in the 34 MET-treated human patients. While the animal models provided high-resolution mechanistic data regarding local neovascularization, macrophage kinetics, and collagen deposition, the clinical findings corroborated that these microenvironmental changes translate directly into superior functional patient recovery, decreased chronic post-operative pain, and enhanced long-term structural stability of the abdominal wall.

## 4. Discussion

The spatial–temporal synchronization of molecular events following prosthetic biomaterial implantation dictates the success of fascial reconstruction. Surgical mesh implantation initiates an immediate plasma protein coating (albumin, fibrinogen, and IgG) and a chemokine-driven leukocyte influx, where early neutrophil-mediated acute inflammation (days 1–3) shifts via the CCL2 pathway into a chronic phase (days 4–14) characterized by macrophage fusion into foreign body giant cells and an IL-4/IL-13-mediated polarization from pro-inflammatory M1 to pro-fibrotic M2 phenotypes. Controlled by the subsequent TGF-β1 and PDGF signaling axes, this microenvironmental equilibrium recruits α-SMA-positive myofibroblasts that dictate the long-term fate of the scaffold, steering the tissue integration trajectory either toward stable Collagen I/III ratio maturation or toward clinical device failure via persistent proteolytic stress and rigid capsule encapsulation.

This scoping review maps evidence from 65 studies to clarify the critical biochemical pivot where a physical scaffold transitions from a persistent immunogenic foreign body to an integrated structural matrix.

A critical finding of this synthesis is the lifelong biochemical activity at the host-mesh interface, challenging traditional paradigms derived from short-term models. While preclinical in vivo animal data frequently suggest microenvironmental stabilization and complete resolution of acute inflammation by week 26 [39,40,41,42,43,44,45], human explant data extending up to 180 months confirm that the foreign body reaction (*FBR*) remains active for decades [46,47,48,49,79,80]. Long-term human explant series demonstrate that polypropylene (*PP*) filaments remain permanently sequestered within active, organized foreign body granulomas. This interface is characterized by a significant, continuous infiltration of CD3+ T-lymphocytes that directly correlates with the density of local CD68+ macrophages (r = 0.341, *p* = 0.001) [48].

This active cellular status demonstrates that the host immune system maintains long-term immunosurveillance rather than achieving true biological inertness. This chronic cellular activation maintains a localized state of proteolytic stress, where more than 50% of the infiltrating macrophages within human tissues continuously co-express Matrix Metalloproteinase-2 (*MMP-2*) [49]. This chronic upregulation drives continuous extracellular matrix (*ECM*) turnover, preventing stable tissue remodeling and weakening the structural integrity of the surrounding fascial scar tissue.

The local inflammatory response at the surgical site initiates a systemic inflammatory cascade that can be quantified through circulating biomarkers. Across the 16 synthesized randomized controlled trials (*RCTs*), C-reactive protein (*CRP*) and Interleukin-6 (*IL-6*) emerged as the definitive circulating markers, showing sharp serum surges within 24 to 48 h post-implantation in 72% of the clinical literature [4,14,15,16,17,18,19,20,21,22,23,24,25,26,27,28,29,30,31,81,82]. This systemic “foreign body signature” is heavily modulated by the choice of surgical access route. Minimally invasive laparoscopic approaches—including transabdominal preperitoneal (*TAPP*) and totally extraperitoneal (*TEP*) techniques—demonstrate a significantly attenuated systemic inflammatory peak compared to open repairs [18,21,25]. This clinical divergence indicates that open repairs cause greater local tissue disruption, releasing high levels of danger-associated molecular patterns (*DAMPs*). These circulating DAMPs amplify signaling through the nuclear factor kappa B (NF-κB) pathway, increasing systemic cytokine output and maintaining an elevated acute-phase response. Over time, continuous exposure to the synthetic polymer prevents the physiological transition from the pro-inflammatory M1 phenotype to the pro-healing M2 phenotype or yields a hybrid, chronically activated macrophage population. Chronic macrophage activation and their subsequent fusion into Foreign Body Giant Cells (FBGCs) maintain a persistent pro-fibrotic environment [80,82].

The structural transition from a localized subclinical reaction to clinical device failure (such as mesh migration, shrinkage, or recurrence) is directly driven by the partial volume of inflammation (*PV-I*) and the partial volume of connective tissue (*PV-CT*). Heavyweight synthetic constructs systematically trigger an aggressive, uncoordinated fibroplastic response. This localized cellular action results in “bridging fibrosis,” where separate fiber-associated granulomas merge into a single, rigid fibrotic plate [50,51,52,53]. This process is mediated by the chronic upregulation of Transforming Growth Factor-beta 1 (*TGF-β1*) at the material interface. High-density polymer fibers alter mechanical tension at the site, forcing local fibroblasts to differentiate into contractile, α-SMA-positive myofibroblasts [54]. This chronic activation downregulates matrix metalloproteinase while upregulating tissue inhibitors of metalloproteinases (*TIMPs*). This biochemical imbalance shifts ECM remodeling toward excessive, rigid Collagen Type I deposition, decreasing abdominal wall compliance and causing chronic groin pain. Conversely, lightweight, large-pore meshes attenuate this tension-sensitive pathway, preserving a balanced Collagen I/III ratio, reducing the total volume of scar tissue, and maintaining physiological elasticity.

Our analysis highlights a substantial translational gap between preclinical models and human clinical realities. Small laboratory animal models (mice and rats) exhibit accelerated metabolic rates and rapid wound-healing kinetics that stabilize the FBR within 90 days, underestimating the long-term immunogenic potential of synthetic polymers in humans by up to 50% [8,41].

Mesh-enriched therapies (*METs*) provide a robust strategy to bridge this translational gap by establishing an active cellular “bio camouflage” over hydrophobic polymer filaments. Biological augmentation using mesenchymal stem cells (*MSCs*), stromal vascular fraction (*SVF*), or autologous hemoderivatives (PRP and PRF) alters the initial host immune response [56,57,58,59,60,61,62,63]. By forcing infiltrating macrophages into an elongated physical morphology, MET upregulates scavenger receptors CD163 and CD206, shifting the local microenvironment away from a pro-inflammatory M1 status and toward a pro-healing, reparative M2 phenotype [66].

This macrophage polarization downregulates microRNA-155, halts the fusion of macrophages into foreign body giant cells (*FBGCs*), and balances the localized IL-6/IL-10 cytokine axis [68]. Furthermore, MET significantly upregulates Vascular Endothelial Growth Factor A (*VEGFA*) and Fibroblast Growth Factor 2 (*FGF2*), driving dense capillary networks through mesh pores. This increased vascularity protects the repair site against late-stage tissue contraction, ischemia, and infectious complications [71,74].

Despite the comprehensive nature of this scoping review, several inherent limitations must be acknowledged. A significant portion of the reviewed literature, particularly within the MET cohort (*n* = 21), relies on small animal models (rats and rabbits) that exhibit accelerated wound healing and distinct immune kinetics, which may not fully replicate the complex, decades-long foreign body reaction observed in human subjects. Furthermore, there is a notable lack of uniformity in the isolation, concentration, and cellular dosages of MET across the 65 included studies, explaining observed discrepancies in outcomes and preventing direct quantitative meta-analyses.

To bridge these gaps, future research in modern herniology must prioritize four pivotal molecular frontiers:

Precision Secretome Therapy: Utilizing isolated exosomes, purified extracellular vesicles, and targeted microRNAs to modulate the canonical TGF-β1/Smad3 pathways with temporal precision, preventing hyper-fibrosis.

Smart Biomaterials: Developing stimuli-responsive, bio-synthetic polymers engineered to release encapsulated growth factors in direct response to localized mechanical strain or microenvironmental pH shifts.

Immune-Niche Engineering: Architecturally modulating the biomaterial surface to engineer a localized immune niche, targeting adaptive immune checkpoints to guarantee long-term graft tolerance and reduce foreign-body giant-cell fusion.

Multi-Omics Validation: Implementing large-scale transcriptomic, proteomic, and single-cell RNA sequencing of long-term human explants to map patient-specific risks and identify predictive circulating biomarkers.

Ultimately, addressing these frontiers through standardized isolation protocols and multi-center clinical registries extending beyond the traditional 2-year surveillance window is paramount to transition the paradigm of hernia repair from a mechanical surgical event to a truly biologically guided therapeutic process.

Based on long-term human explant data (up to 180 months) and experimental biological augmentation, we propose the following recommendations:

- **Optimizing Mesh Selection (“Less is More”)**: To minimize chronic inflammation (PV Inflammation), surgeons should prioritize lightweight (LW), large-pore meshes. Data confirms that LW designs reduce the risk of “bridging fibrosis” and preserve long-term abdominal wall compliance compared to heavyweight options.
- **Strategic Integration of MET:** Clinical consideration should be given to biological augmentation (e.g., PRP, growth factor gels) to accelerate the M1-to-M2 macrophage phenotypic shift. This approach enhances local vascularization (VEGF expression), potentially preventing tissue ischemia and mesh erosion.
- **Biomarker-Guided Monitoring:** In high-risk cohorts (e.g., recurrent hernias, diabetes, heavy smoking), monitoring matrix remodeling indicators (MMP2, TNF-α) may provide early warning signs of pathological integration. While cost-intensive, standardized molecular screening could preempt chronic pain or mechanical failure.
- **Calibrating the “Biological Window”:** To avoid TGF-β1-induced hyper-fibrosis, enrichment therapies must be standardized. We recommend precise concentrations (e.g., PRP 4-5 fold baseline) to ensure tissue elasticity is not compromised by excessive scarring.
- **Conservative Management of Late-Stage Complications:** Clinicians must recognize the periprosthetic granuloma as a dynamic entity. In cases of minor erosion or chronic irritation, localized regenerative therapies (PRP infiltrations, CO2 laser) may serve as conservative alternatives to radical surgical explantation.
- **Lifelong Surveillance and Registries:** As the FBR persists for decades, clinical follow-up must extend beyond the standard 1–2-year window. Long-term registries are essential to evaluate lifelong polymer degradation and the true stability of the prosthetic–host interactome.

## 5. Conclusions

This scoping review, synthesizing data across 65 heterogeneous studies, underscores a fundamental paradigm shift in modern prosthetic hernia surgery: the transition from managing a passive, destructive foreign body reaction (*FBR*) to orchestrating proactive, cell-mediated biological integration. The extracted evidence proves that standard synthetic meshes are not biologically inert but remain biochemically active within the host for decades. Left unmodulated, heavyweight polypropylene constructs drive chronic inflammation, local proteolytic stress via sustained MMP-2 upregulation, and non-compliant bridging fibrosis that clinically manifests as chronic post-operative groin pain and device structural failure.

The clinical emergence of mesh-enriched therapies (*MET*) provides a crucial “biological bridge” capable of reprogramming this hostile microenvironment. By creating an active cellular biocamouflage layer using mesenchymal stem cells (*MSCs*), stromal vascular fraction (*SVF*), or autologous hemoderivatives like PRP and PRF, MET actively redirects the local inflammatory cascade away from an aggressive M1 macrophage phenotype and toward a pro-healing, regenerative M2 status. This immunomodulatory pivot optimizes the localized IL-6/IL-10 cytokine axis, enhances VEGF-driven capillary neovascularization, stabilizes the structural Collagen I/III ratio, and preserves native abdominal wall compliance. Ultimately, the synergy between advanced synthetic scaffolds and targeted biological agents offers transformative clinical potential to simultaneously minimize long-term recurrence rates and chronic pain.

However, successful bench-to-bedside translation is currently hindered by a substantial translational gap and reporting inconsistencies. Small animal models with accelerated metabolic profiles underreport the lifelong immunogenic risks of polymers by up to 50%, while a lack of standardization in cell dosages and growth factor release kinetics risks triggering hyper-fibrosis rather than functional integration. Future clinical success relies on the manufacturing of smart, stimuli-responsive, and controlled-release matrices capable of precision biological delivery. Overcoming these barriers will require the harmonization of isolation protocols and the establishment of international, multi-center patient registries extending well beyond the traditional 2-year surveillance window to guarantee that a foreign body encounter is permanently transformed into a seamless, lifelong integration with the human host.

## Figures and Tables

**Table 1:** Methodological mapping, study characteristics, and biomarker profiles of the included studies (*N* = 65).

Study Category/Design	Model and Sample Size	Evaluated Biomaterials and Surgical Approaches	Primary Evaluated Markers	Key Clinical and Biological Observations
Systemic Response RCT (*n* = 16) Prospective (*n* = 3) Retrospective (*n* = 6)	Human cohorts (*n* = 1222 patients)	Polypropylene (PP) mesh (*n* = 23 studies); Open vs. Laparoscopic approaches	CRP (72%), IL-6 (72%), WBC count (40%), TNF-α (24%)	Mesh implantation triggers a predictable “foreign body signature” with acute CRP and IL-6 surges within 48 h post-surgery. Minimally invasive techniques (TAPP/TEP) demonstrate significantly attenuated systemic inflammatory peaks compared to extensive open tissue-disrupting repairs.
Local FBR Dynamics Human Explants (*n* = 4) In Vivo Animal (*n* = 13) In Vitro/Ex Vivo (*n* = 2)	743 recovered meshes; 674 animals (rats, rabbits, mice)	Heavyweight (HW) vs. Lightweight (LW) PP meshes; Timeline: 1 day to 180 months	PV of Inflammation, PV of Connective Tissue, M1/M2 Ratio, MMP-2, Ki67/TUNEL	The foreign body reaction is a lifelong process, not a transient event, as confirmed by human explants up to 15 years. Heavyweight meshes trigger aggressive bridging fibrosis via canonical TGF-β1/Smad3 signaling, driving contractile α-SMA+ myofibroblast differentiation and non-compliant scar plates.
MET Integration Preclinical (*n* = 19) Clinical Translation (*n* = 2)	514 animal subjects; 34 MET-treated human patients	Autologous cells (MSCs, SVF, ADSCs) or hemoderivatives (PRP, PRF) on scaffolds	CD163+, CD206+ (M2 markers), VEGFA, FGF2, CD90/CD44/CD45	Biological augmentation using cellular shrouds or blood-derived secretomes shifts the host immune profile toward a pro-regenerative M2 phenotype. This immunomodulatory pivot consistently enhances VEGF-driven neovascularization and optimizes the structural Collagen I/III ratio.

## Data Availability

No new data were created or analyzed in this study. Data sharing is not applicable to this article.

## Supplementary Materials

The following supporting information can be downloaded at: https://www.mdpi.com/article/10.3390/ijms27146153/s1.

## Abbreviations

The following abbreviations are used in the manuscript:

ADSCs	Adipose-Derived Stem Cells
ANG-1	Angiopoietin-1
CD	Cluster of differentiation
CO2	Carbon dioxide
CRP	C-reactive protein
ECM	Extracellular matrix
EMT	Epithelial–Mesenchymal Transition
FBGCs	Foreign Body Giant Cells
FBR	Foreign Body Responses
FGF-2	Fibroblast Growth Factor 2
GAPDH	Glyceraldehyde 3-phosphate dehydrogenase
IFN-γ	Interferon gamma
IHC	Immunohistochemistry
IL	Interleukin
LW	Lightweight
M1 macrophage	Pro-inflammatory macrophage
M2 macrophage	Anti-inflammatory macrophage
MeSH	Medical Subject Headings
MET	Mesh-Enhanced Therapies
MMP-2	Matrix metalloproteinase-2
mRNA	Messenger Ribonucleic Acid
MSCs	Mesenchymal stem cells
OSF	Open Science Framework
PCC	Population, Concept, Context
PP	Polypropylene
PRF	Platelet-Rich Fibrin
PRISMA-ScR	Preferred Reporting Items for Systematic reviews and Meta-Analyses extension for Scoping Reviews
PRP	Platelet-rich plasma
PV	Partial Volume
RCT	Randomized Controlled Trial
SVF	Stromal vascular fraction
TAPP	Transabdominal Preperitoneal
TEP	Totally Extraperitoneal
TGF-β1	Transforming Growth Factor beta 1
Th	T helper
TNF-α	Tumor Necrosis Factor alpha
TUNEL	Terminal deoxynucleotidyl transferase dUTP Nick End Labeling
VEGF	Vascular Endothelial Growth Factor
WBC	White Blood Cell
α-SMA	Alpha-Smooth Muscle Actin
