# Supplementary material for: Mapping the Prosthetic–Host Interactome: From Systemic Inflammation to Biological Integration in Mesh-Enhanced Therapies METs—A Scoping Review
Source: Int J Mol Sci. 2026 Jul 9;27(14):6153. doi: 10.3390/ijms27146153 (PMC13409846; doi:10.3390/ijms27146153)
Supplement: Supplementary file 1 [file ijms-27-06153-s001.zip › ijms-4355705-supplementary.pdf]

Database	Interface / Platform	Full Search String Syntax
**PubMed**	NCBI	(("Hernia, Inguinal"[MeSH] OR "Hernia, Femoral"[MeSH] OR "Groin Hernia*"[Title/Abstract])) AND (("Surgical Mesh"[MeSH] OR "Mesh*"[Title/Abstract] OR "Prosthesis"[Title/Abstract])) AND (("Mesenchymal Stem Cells"[MeSH] OR "Cell- and Tissue-Based Therapy"[MeSH] OR "Stem Cell*"[Title/Abstract] OR "Stromal Vascular Fraction"[Title/Abstract] OR "Bio-synthetic"[Title/Abstract] OR "Cell-Enriched"[Title/Abstract] OR "MET"[Title/Abstract]))
**Embase**	Elsevier	('groin hernia'/exp OR 'groin hernia*':ti,ab OR 'inguinal hernia'/exp OR 'femoral hernia'/exp) AND ('surgical mesh'/exp OR 'mesh*':ti,ab OR 'prosthesis':ti,ab) AND ('mesenchymal stem cell'/exp OR 'cell therapy'/exp OR 'stem cell*':ti,ab OR 'stromal vascular fraction':ti,ab OR 'bio-synthetic':ti,ab OR 'cell-enriched':ti,ab OR 'met':ti,ab)
**Scopus**	Elsevier	(TITLE-ABS-KEY("groin hernia*") OR TITLE-ABS-KEY("inguinal hernia") OR TITLE-ABS-KEY("femoral hernia")) AND (TITLE-ABS-KEY("surgical mesh") OR TITLE-ABS-KEY("mesh*") OR TITLE-ABS-KEY("prosthesis")) AND (TITLE-ABS-KEY("mesenchymal stem cell*") OR TITLE-ABS-KEY("stem cell*") OR TITLE-ABS-KEY("stromal vascular fraction") OR TITLE-ABS-KEY("met"))

Supplementary Table S1. Comprehensive Search Strategies Adapted for Each Interrogated Database.

Supplementary Table S2. Methodological Quality Assessment and Risk of Bias Summary for Included Studies (N = 65).

Study Cohort / Category	Assessment Tool Applied	Evaluated Domains / Parameters	Overall Methodological Quality & Risk of Bias Summary
**Systemic Footprint** (n = 25 studies) • *Human clinical trials & RCTs*	**JBI (Joanna Briggs Institute)** Critical Appraisal Checklists for Randomized Controlled Trials and Cohort Studies	• Randomization integrity • Allocation concealment • Blinding of patients/assessors • Baseline homogeneity • Outcome measurement reliability	**Low Risk of Bias (High Quality):** Over 80% of the clinical trials met all core JBI reporting standards. Patient selection and outcome parameters were clearly defined. Blinding was well-maintained in 12 out of 16 RCTs.
**Local FBR Dynamics** (n = 19 studies) • *Human explants & In vivo models*	**JBI & SYRCLE** Systematic Review Center for Laboratory Animal Experimentation Risk of Bias Tool	• Baseline characteristics matching • Sequence generation (animal) • Allocation concealment • Animal housing randomization • Blinding of outcome assessors	**Moderate Risk of Bias:** Retrospective human explant studies exhibited high consistency in tissue processing but lacked early clinical baseline data. *In vivo* animal studies suffered from poor reporting of assessor blinding (unreported in 68% of studies).
**MET Integration** (n = 21 studies) • *Preclinical & Translation*	**SYRCLE & JBI** Animal Risk of Bias Framework & JBI Case Series Checklists	• Selection bias (sequence generation) • Performance bias (housing) • Detection bias (blinding) • Attrition bias (incomplete data)	**Moderate to Low Risk of Bias:** Stem cell and blood-derived (PRP/PRF) validation protocols (CD markers, viability assays) were highly rigorous. However, randomization sequence details during animal allocation were frequently omitted.
